# The Impact of Heritable Myopathies on Gastrointestinal Skeletal Muscle Function

**DOI:** 10.1016/j.jcmgh.2025.101522

**Published:** 2025-04-22

**Authors:** Aishwarya Iyer, Madeline Alizadeh, Jennifer Megan Mariano, Aikaterini Kontrogianni-Konstantopoulos, Jean-Pierre Raufman

**Affiliations:** 1 Department of Biochemistry and Molecular Biology, University of Maryland School of Medicine, Baltimore, Maryland; 2 The Institute for Genome Sciences, University of Maryland School of Medicine, Baltimore, Maryland; 3 Department of Medicine, Division of Gastroenterology and Hepatology, University of Maryland School of Medicine, Baltimore, Maryland; 4 VA Maryland Healthcare System, Baltimore, Maryland; 5 Marlene and Stewart Greenebaum Cancer Center, University of Maryland School of Medicine, Baltimore, Maryland

**Keywords:** Dysphagia, GI Tract Skeletal Muscle, Hereditary Myopathies

## Abstract

Among other contributions to gastrointestinal (GI) function, skeletal muscles regulate transit at both ends of the GI tract by providing propulsive forces for ingested nutrients and controlling the excretion of waste products. At the oropharynx, skeletal muscles provide necessary forces for effective mastication and the transfer of food boluses from the mouth into the proximal esophagus, where skeletal muscle-mediated peristalsis initiates propulsion of food boluses towards the stomach, a function supplanted by the upper esophagus smooth muscle. Consequently, the most prominent manifestation of proximal GI tract skeletal muscle dysfunction is transfer and oropharyngeal dysphagia that may result in repeated episodes of life-threatening choking and pulmonary aspiration. At the anal canal, the external anal sphincter controls the release of gas, liquids, and solids. Skeletal muscles within the pelvic floor play a synergistic role in regulating defection. Hence, distal GI tract skeletal muscle dysfunction may result in the leakage of flatus and fecal matter, whereas, in contrast, pelvic floor dysfunction may contribute to constipation. The balance between such defects may severely impact nutritional status and quality of life. Herein, we provide a comprehensive review of the genetics, molecular biology, and mechanisms underlying heritable disorders of skeletal muscle and how these may impact GI tract function and overall well-being. For organizational purposes, we separate discussions of congenital, mitochondrial, and myofibrillar myopathies and muscular dystrophies. For the sake of completeness, we also briefly consider acquired myopathies that affect GI tract function. As treatment options are currently limited, disorders of skeletal muscle function provide exciting therapeutic opportunities, including innovative approaches to target specific gene modifications.

**Summary**

Heritable defects of skeletal muscle genes, key players in the proximal and distal gastrointestinal tract, may adversely affect nutrition and well-being. We review the genetics and molecular biology underlying these disorders, and current approaches to managing resultant adverse gastrointestinal effects.

Characterized histologically by alternating dark and light bands resulting in characteristic striations, skeletal muscles provide propulsive forces for ingested nutrients and facilitate the excretion of waste at the proximal and distal ends, respectively, of the luminal gastrointestinal (GI) tract (Figure 1). Accordingly, skeletal muscle plays a key role in digestive health and disease. Skeletal muscles in the oral cavity (eg, the tongue), oropharynx, and proximal esophagus provide the force for effective mastication and swallowing, while protecting the airway, and propel food boluses from the mouth into the proximal esophagus where they initiate peristalsis. At the intestinal terminus, the external anal sphincter, in concert with striated musculature in the pelvic floor, controls the release of intestinal gas and fecal matter. Among other disorders, dysfunction of proximal GI tract skeletal muscle may result in transfer dysphagia, wherein the delivery of food to the esophagus and stomach is delayed or misdirected, and choking and pulmonary aspiration are provoked. External anal sphincter dysfunction may incite uncontrollable leakage of flatus and stool. In contrast, dysfunction of skeletal muscles comprising the pelvic floor may result in constipation. Thus, the balance between external anal sphincter and pelvic floor dysfunction may modify the clinical manifestations. Given these central roles in maintaining GI homeostasis, skeletal myopathies have the propensity to severely impact nutrition and quality of life. Moreover, as current treatment options are limited, disorders of skeletal muscle function provide novel therapeutic opportunities, including new gene-editing approaches applied to other diseases.^1^

**Figure 1:**
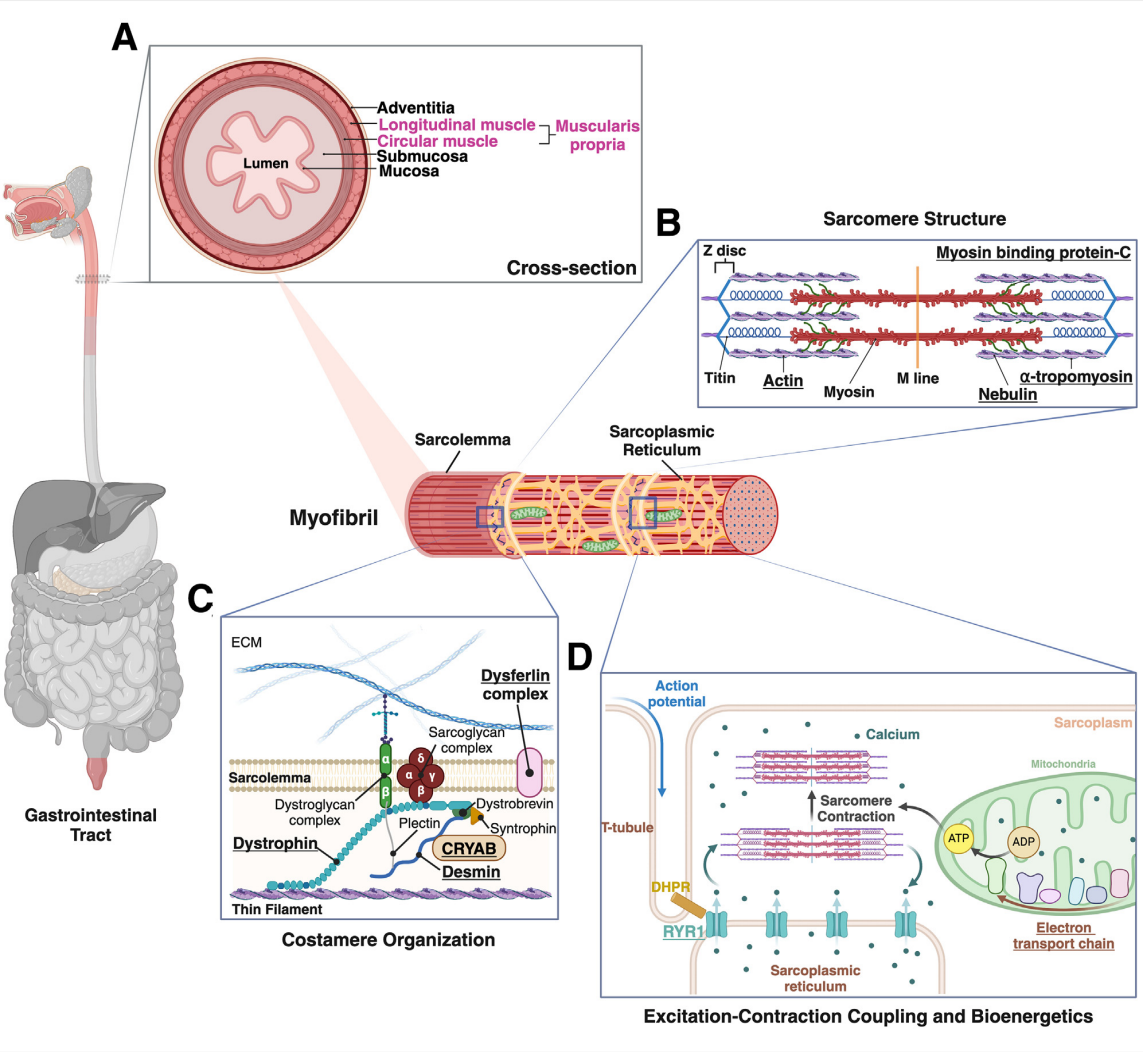
**Distribution and function of skeletal muscle in the human GI tract.** As depicted in *pink* in the anatomical rendition of the GI tract (*left*), skeletal muscle is located primarily at the entrance (eg, tongue, proximal esophagus) and exit (eg, anus) of the GI tract (*red and pink regions*). (*A*) Within the luminal GI tract, skeletal muscle is located primarily within the longitudinal and circular muscle layers. The central graphic depicts a representative myofibril showing the 3-dimensional orientation of sarcomeres and the sarcoplasmic reticulum. Inset (*B*) depicts the representative ultrastructure of a sarcomere, the basic functional unit within a myofibril that allows for organized muscle contraction and relaxation. Inset (*C*) illustrates a simplified schematic of the costamere, a sarcolemma-sarcomere connector essential in maintaining structural integrity of muscle cells during contraction-relaxation. Inset (*D*) portrays an overview of excitation-contraction coupling, responsible for calcium-induced activation of muscle contraction following nerve impulses, and mitochondrial bioenergetics necessary to efficiently generate energy for muscle contraction. All these features are essential components of physiological muscle function. Created with BioRender.com.

With recent advances in the mechanistic understanding of the genetic mutations underlying heritable myopathies, it is timely to review their impact on GI tract function. Herein, we review the gross, microscopic, and cellular anatomy of skeletal muscle, and describe how forces generated by skeletal muscle in the proximal and distal GI tract modulate normal function. Then, we describe how mutations in relevant proteins can modify cell and organ function and outline the resulting clinical ramifications. As we focused this review on the GI tract, we refer readers to our reference list for topics omitted from this review and for those covered in greater detail by others; to guide the reader, we highlighted relevant references at appropriate places in the text.

## Skeletal Muscle in the GI Tract

Although skeletal muscle is the predominant muscle type in the oropharynx (eg, tongue and cricopharyngeal muscles) and the pelvic floor, it is generally restricted to the proximal esophagus and distal rectum within the luminal GI tract (Figure 1).^2^ The remainder of the GI tract, extending from the upper/mid-esophagus to the internal anal sphincter, is comprised of smooth muscle. Between this transition zone and the rectum, the musculature of the luminal GI tract is organized within circular and longitudinal layers in the muscularis externa (Figure 1*A*).

Skeletal muscles in the mouth and proximal esophagus are crucial for mastication and deglutition of solids and liquid foods during distinct phases: oral preparatory, oral transport, pharyngeal, and esophageal phases.^3^ Impairment of any phase can result in dysphagia. Weakening of the tongue affects most phases, as the tongue’s actions are essential for physical mechanisms maintained in the oral preparatory phase—food bolus formation in the oral transport phase and oropharyngeal transport of the bolus in the pharyngeal phase. Notably, proper oral preparatory phase mechanisms are vital for neonatal nutrition, evidenced by the common need for nasogastric tube feeding in infants with congenital myopathies.^3^ Furthermore, oral and pharyngeal muscle coordination are essential to prevent aspiration and choking, a common occurrence in myopathies resulting in aspiration pneumonia. Abnormal upper esophageal sphincter function and proximal esophageal dysmotility can impede the esophageal phase, which in addition to dysphagia, may manifest as laryngopharyngeal reflux.^3^^,^^4^

The external anal sphincter, the sole region in the distal luminal GI tract composed of skeletal muscle, regulates defecation in concert with rectal motor activity and the internal anal sphincter^5^ (Figure 1). Impaired external sphincter muscle function impacts fecal continence,^6^ which has been observed in myotonic dystrophy.^7^^,^^8^ Yet, in contrast to upper GI manifestations, fecal incontinence is rarely reported in patients with myopathies. Several possibilities may account for this surprising observation—the muscles comprising the external anal sphincter may be less affected, potentially due to compensatory actions by the smooth muscle-comprised internal anal sphincter,^9^ fecal incontinence may simply be under-reported or minimally investigated, or opposing effects of pelvic floor skeletal muscle dysfunction promoting constipation may compensate for the effects of external sphincter dysfunction.

## Skeletal Muscle Organization

To effectively execute muscle contraction-relaxation, myofibrils, the basic building block of skeletal myofibers, are composed of a cytoskeletal network of proteins, occupying repeating sarcomere units.^10^ Heritable myopathies impacting GI function result from mutations in proteins important for 4 major aspects of myofibril structure and function: sarcomere structure, costamere organization, bioenergetics, and excitation-contraction coupling (Figure 1).

Sarcomeres are composed of essential thick and thin filaments, whose interactions are integral for actomyosin crossbridge cycling and subsequent muscle contraction^10^ (Figure 1*B*). Mutations in thin filament proteins (eg, actin, tropomyosin, nebulin) can result in congenital myopathies associated with feeding difficulties.^11^^,^^12^ Slow skeletal Myosin Binding Protein-C (sMyBP-C), an essential accessory protein localized to the sarcomeric A-band, interacts with thick and thin filament proteins to regulate crossbridge cycling; sMyBP-C mutations are linked to arthrogryposis syndromes and a myopathy associated with a characteristic tremor phenotype.^13^, ^14^, ^15^, ^16^

Costameres, located at the Z-disk, provide both mechanical and signaling functions, bridging from the sarcolemma to the sarcomere to the extracellular matrix, that maintain myofibril structural integrity during cycles of muscle contraction and relaxation^17^ (Figure 1*C*). Due to dystrophin’s interactions with sarcomeric actin and membranous β-dystroglycan, the dystrophin-glycoprotein complex is essential for costamere structure and function. Mutations in dystrophin may result in Duchenne or Becker muscular dystrophy.^17^ The essential intermediate filament protein, desmin, also maintains cytoskeletal organization within the muscle fiber. Although not a core component of the dystroglycan complex, desmin links the sarcomere to the sarcolemma and essential organelles (eg, nuclei and mitochondria), while also promoting lateral z-disk connectivity between myofibrils.^18^ Mutations in desmin and desmin-associated proteins (ie, CRYAB) also result in myofibrillar myopathies.^19^^,^^20^

Excitation-contraction coupling is the mechanism whereby a neural-evoked action potential leads to voltage-dependent calcium release in muscle cells, which induces muscle contraction (Figure 1*D*). Sarcolemmal dihydropyridine receptors (DHPRs) detect depolarization, while ryanodine receptors (RYR1s) in the sarcoplasmic reticulum release calcium ions that subsequently bind to troponin in the thin filament. This series of events fosters actomyosin crossbridge formation and sarcomere shortening.^21^ Congenital myopathies may result from impaired excitation-contraction coupling due to RYR1 mutations.^12^^,^^22^ Calcium also plays an integral role in regulating mitochondrial dynamics and signaling, which is necessary for skeletal muscle metabolic function and plasticity.^23^^,^^24^ Mitochondrial dysregulation via mitochondrial DNA mutations can impede oxidative phosphorylation, resulting in mitochondrial myopathies.^24^

## Effects of Heritable Myopathies on GI Tract Skeletal Muscle Function

An overview of the genetics, structural consequences, and GI manifestations of hereditary skeletal myopathies is provided in Table 1.

**Table 1:** Genetics, Structural Consequences, and GI Manifestations of Hereditary Skeletal Myopathies

Category	Myopathy	Incidence	Affected genes (inheritance pattern)	Structural consequences	GI manifestations	References
Congenital myopathies	NM	1:50,000 births^a^	Mutations in ≥12 genes encoding sarcomeric proteins – *NEB, ACTA1, TPM3, TNNT1, KLH41,* etc. (variable inheritance)	Altered thin filament structure and regulation	Dysphagia, pyloric stenosis, GERD (congenital); achalasia and chronic upper GI tract pseudo-obstruction (childhood-onset); pneumatosis cystoides intestinalis	11, 25, 26, 27, 28, 29
	XLMTM	17:1 million births	*MTM1* mutation (X-linked)	Altered membrane trafficking, cytoskeletal organization, and muscle regeneration	Dysphagia, pyloric stenosis, GI bleeding	30, 31, 32
	Severe congenital RYR1-associated myopathy	Unknown	*RYR1* mutation (AD and AR)	Impaired excitation-contraction coupling	Feeding difficulties	22
	CFTD	Unknown	*TPM3, RYR1, ACTA1, TPM2* mutations (variable inheritance)	Altered TPM3 binding to actin (TPM3-related CFTD)	High-arched palate, dysphagia	12, 33, 34
	Myotrem myopathy	Unknown	Mutations in *MYBPC1* (AD)	Altered crossbridge cycling and myosin/actin binding	Poor sucking and feeding during infancy, tongue tremor, high-arched palate, dysphagia	13, 14, 35, 36, 37, 38
	DA	1:20,000 births	Mutations in *MYBPC1, TNNI2, TNN3, TPM2, MYH3,* and *MYH8* (AD, AR)	Likely interplay between early muscle development and limited fetal movement *in utero*	Poor feeding, impaired suckling and swallowing	39, 40, 41, 42, 43, 44, 45, 46, 47
Mitochondrial myopathies	MERRF	Highly mutation dependent (MDDs generally affect 1:5000 persons)^a^	Mitochondrial DNA mutations (most often in *MT-TK*)	Dysfunctional mitochondrial functions, including diminished ATP production, impaired reactive oxygen species scavenging, and compromised calcium homeostasis, impairs tissue metabolism	Dysphagia, pancreatitis, hepatopathy, intestinal pseudo-obstruction	24, 48, 49, 50, 51, 52, 53, 54
	MNGIE		*TYMP* mutations (AR)		Poor appetite, dysphagia, vomiting, gastroesophageal sphincter dysfunction (reflux), gastroparesis and delayed emptying, intestinal pseudo-obstruction and diverticulosis, constipation, diarrhea	
	KSS		MtDNA deletions and duplications		Dysphagia, vomiting, gastroesophageal sphincter dysfunction (cricopharyngeal achalasia), pancreatitis	
	PEO - PEO3, PEO5		MtDNA deletions		Dysphagia, pancreatitis, cricopharyngeal achalasia, hepatopathy	
Myofibrillar myopathies	Desminopathy	Unknown	Mutations in *DES*	Likely disrupted intermediate filament network	Dysphagia, dysphonia, vomiting, constipation	19, 20, 50
	Alpha-B crystallin myopathy	Unknown	Mutations in *CRYAB* (AD or AR)	Postulated to alter sarcomere Z-band structure and skeletal muscle homeostasis	Dysphagia, dysphonia, high arched palate	19, 55, 56, 57, 58
	Myotilinopathy/ LGMD1A	Unknown	Mutations in *MYOT* (AD)	Potentially compromised sarcomere structural integrity due to myotilin aggregation	Dysarthria	59, 60, 61
Muscular dystrophies	DMD	1:3500 to 1: 5000 live male births	Mutation in *DMD* (X-linked recessive)	Loss of dystrophin function disrupts costamere signaling and macromolecular interactions that maintain cytoskeletal structure impair myofibril function and eventually lead to muscle degeneration	Dysphagia, GERD, macroglossia, constipation, pneumatosis cystoides intestinalis, weight loss	27, 62, 63, 64, 65, 50
	BMD	1:30,000 male births			Mastication difficulties, dysphagia	66, 67, 68
	OPMD	Prevalence of 1:100,000^a^	Expansion repeats of *PABPN1* (AD or rarely AR)	Likely protein aggregation in cell nuclei impairs cell function	Progressive dysphagia (late-onset), esophagitis	69, 70, 71, 72
	DM – DM1 and DM2	1:1200 (DM1) ≥1:8000^a^ (DM2)	MD1: expansion of CTG repeats in *DMPK* (AD) MD2: intronic CCTG repeats in *CNBP*	The excess nucleotide repeats result in transcribed RNA promotes sequestration of RNA-binding proteins and dysregulation of RNA splicing	Dysphagia, diarrhea, abdominal pain, constipation, intestinal pseudo-obstruction and volvulus, delayed gastric emptying, gallbladder dysfunction, GERD, fecal incontinence	7, 8, 73, 74, 75, 76, 77, 78, 79, 80, 81, 82, 83
	FSHD-1	Prevalence 1:20,000	Contraction of D4Z4 repeat array at chr4q35.2 (AD)	Hypomethylation of the D4Z4 region and subsequent de-repression of *DUX4* in striated muscle	Dysphagia resulting from tongue atrophy and jaw muscle weakness	84, 85
	Non-myofibrillar LGMD (heterogeneous clinical subtypes)	1:100,000 (all forms)	Mutations in *DNAJB6* (LGMD1D), *TNPO3* (LGMD2D), *DYSF* (LGMD2B), etc. (AD or AR inheritance is subtype-dependent)	Sarcomere instability (ie, dystrophin-glycoprotein complex) with impaired protein trafficking and turnover	Dysphagia	86, 87, 88
Miscellaneous myopathies	EMARDD	Unknown	Mutations in *MEGF10* (AR)	Likely due to impaired myogenesis from altered *MEGF10* expression in muscle satellite cells	Oral cavity structural abnormalities, dysphagia	89, 90, 91, 92
	ADSSL1 myopathy	Unknown	*ADSSL1* mutations (AR)	Striated muscle fat infiltration and impaired ATP-generating enzymes	Easy fatigability, dysphagia with masticatory dysfunction	93, 94, 95

ATP, adenosine triphosphate; AD, autosomal dominant; AR, autosomal recessive; BMD, Becker’s muscular dystrophy; CFTD, congenital fiber-type disproportion myopathy; DA, distal arthrogryposis; DMD, Duchenne muscular dystrophy; DM, myotonic dystrophy; EMARDD, early-onset myopathy, areflexia, respiratory distress, and dysphagia; FSHD, facioscapulohumeral dystrophy; GERD, gastroesophageal reflux disease; GI, gastrointestinal; LGMD, limb girdle muscular dystrophy; KSS, Kearns-Sayre syndrome; MNGIE, mitochondrial neurogastrointestinal encephalomyopathy; MERRF, myoclonic epilepsy and ragged red fibers; NM, nemaline myopathy; OPMD, oculopharyngeal muscular dystrophy; PEO, progressive external ophthalmoplegia; XLMTM, X-linked myotubular myopathy.

a Estimated incidence can vary depending on studied population, communities, and countries.

### Congenital Myopathies

Congenital myopathies manifest with hypotonia and muscle weakness at birth or infancy, whereas symptom progression is commonly limited or slow.^25^ Five myopathy subtypes are recognized—nemaline, core, centronuclear, congenital fiber-type disproportion, and myosin storage.^25^ Here, we consider only subtypes resulting in GI manifestations.

Nemaline myopathy (NM) encompasses heterogeneous disorders with varying patterns of inheritance, disease onset, progression, and severity. Classical manifestations of muscle weakness and hypotonia are common across all clinical subtypes.^26^ These disorders are predominantly caused by mutations in thin filament proteins, most commonly nebulin (*NEB*) and ⍺-actin (*ACTA1*). Altered structure and function of the thin filament, leading to impaired actomyosin crossbridge formation, is associated with muscle weakness.^26^ However, the exact mutation-specific mechanisms underlying NM pathophysiology remain to be determined. Due to profound weakness of orofacial and oropharyngeal muscles, GI involvement in congenital NM impairs sucking and swallowing, often requiring nasogastric or gastrostomy tube feeding to provide adequate nutrition.^11^^,^^96^ Subsequent reduced food intake can promote malnutrition and recurrent aspiration-induced pulmonary infections.^96^ Rectal wall pneumatosis cystoides intestinalis may result in episodes of constipation and hematochezia.^27^ As NM also affects the GI tract smooth muscle, gastroesophageal reflux and pyloric stenosis may occur. In adolescents with childhood-onset NM, dysphagia and chronic upper intestinal pseudo-obstruction may be seen.^11^^,^^97^

X-linked myotubular myopathy (XLMTM) results from mutations in myotubularin (*MTM1*), a phosphoinositide phosphatase important for myocyte homeostasis, including autophagy and vesicular transport.^28^ *MTM1* mutations impair membrane trafficking, excitation-contraction coupling, and muscle regeneration, resulting in neonatal hypotonia and particularly profound muscle weakness in males.^28^ Severe respiratory insufficiency may result from dysphagia, requiring gastrostomy tube placement. Notably, female XLMTM carriers can also develop progressive myopathic symptoms with variable dysphagia onset and severity.^28^^,^^30^

Severe congenital *RYR1*-associated myopathy, a central core myopathy, results from mutations in the gene encoding the skeletal muscle RYR1 channel. *In utero* consequences include polyhydramnios and decreased fetal movement. In addition to markedly decreased muscle tone and strength, infants have contractures, skeletal deformities, dysmorphic features, respiratory distress, and feeding difficulties.^22^^,^^98^ Recessive mutations are associated with increased disease severity, whereas dominant mutations commonly have single amino acid substitutions in the conserved N-terminal and C-terminal regions.^22^ Mutation-dependent effects on RYR1 function alter intracellular calcium release, potentially explaining the wide range of mutation-dependent severity and how abnormal excitation-contraction coupling impairs muscle function.^22^^,^^99^

Congenital fiber-type disproportion myopathy (CFTD) is associated with progressive generalized muscle weakness, hypotonia, respiratory impairment, and dysphagia.^12^^,^^33^ The most common causes include mutations in *RYR1*, *ACTA1*, and slow ⍺-tropomyosin (*TPM3*), the latter being the most prevalent. *TPM3* mutations impair TMP3 dimerization and stability, thereby altering interactions with actin.^34^

Myotrem, a myopathy recently identified by our group and collaborators, is caused by pathogenic variants in *MYBPC1*, the gene encoding sMyBP-C.^13^^,^^14^^,^^35^, ^36^, ^37^, ^38^ Affected individuals have early-onset muscle weakness, congenital hypotonia, skeletal deformities, respiratory insufficiency, and a characteristic tremor of myogenic origin, in the absence of neuropathy.^13^^,^^14^^,^^35^, ^36^, ^37^, ^38^ This tremor affects the tongue and chin,^13^^,^^14^^,^^35^^,^^37^ as well as the upper and lower extremities, is typically present since birth, and is exacerbated upon intention, action, or posture.^13^^,^^14^^,^^35^^,^^37^^,^^38^ Many individuals report early-life feeding difficulty and dysphagia, sometimes requiring tube feeding, that gradually resolve in childhood.^13^^,^^35^ This may be related to anatomical abnormalities present at birth, including high-arched palate,^14^^,^^35^ micrognathia,^14^ and cleft lip^37^ that impede effective swallowing mechanisms. As a result of reduced nutritional intake, Myotrem individuals generally have lean mass and minimal body fat. A murine Myotrem model carrying the dominant E248K pathogenic variant exhibited embryonic lethality when inherited in a homozygous manner,^14^ and contractile deficits, skeletal deformities, and decreased body mass with heterozygous inheritance.^100^

Distal arthrogryposis (DA), a subtype of arthrogryposis multiplex congenita, observed in 1 per 3000 live births, has a heterogenous clinical phenotype characterized by congenital contractures in distal joints.^39^ These contractures, thought to result from limited fetal movements *in utero*, are associated with mutations in multiple sarcomeric genes including *TNN3*, *TPM2*, *MYH8*^39^, and *MYBPC1*.^40^ Variation in clinical phenotypes gives rise to at least 10 subtypes^39^—the most common, DA type I (DA1), is characterized by camptodactyly, clubfoot, overlapping fingers, hypoplasia in finger creases, and micrognathia.^41^ DA type 2A (DA2A), Freeman-Sheldon syndrome, has a distinctive “whistling-face” phenotype of a small oral orifice with puckered lips and an H-shaped dimple on the chin.^42^ Dental crowding resulting from these facial deformities, recurrent vomiting, and feeding difficulties commonly require orogastric or nasogastric tube feedings.^42^ DA type 2B (DA2B), Sheldon-Hall syndrome, is similar but less severe than DA2A with micrognathia and high arched, cleft lip palates.^43^ DA type 3 (DA3), Gordon syndrome, is characterized by short stature and cleft palate and/or bifid uvula.^44^ DA type 7 (DA7), Hecht-Bael syndrome, is characterized by trismus, usually leading to feeding difficulties, and joint flexion deformities (pseduocamtodactyly).^45^ We recently reported compound heterozygote mutations in *MYBPC1*, leading to a DA1-like myopathy with muscle weakness, feeding difficulties requiring nasogastric tube feeding, and spinal rigidity with tremor.^46^ Diagnostic criteria for other DA subtypes include ocular and/or respiratory abnormalities.^39^

### Mitochondrial Myopathies

Mitochondrial myopathies (MIDs) result from mutations in mitochondrial DNA.^48^ For example, mutations in genes encoding mitochondrial transfer RNA (tRNA), commonly tRNA^Lys^, result in myoclonic epilepsy and ragged red fibers (MERRF), characterized by myoclonus, seizures, ataxia, and muscle weakness.^24^ Mutations in thymidine phosphorylase cause excess accumulation of toxic metabolites and mitochondrial neurogastrointestinal encephalomyopathy (MNGIE) characterized by progressive deterioration of the GI tract.^24^ Both MERRF and MNGIE can present with dysphagia from combined oropharyngeal muscle weakness and neural impairment.^48^ Large mitochondrial DNA deletions are characteristic of chronic progressive external ophthalmoplegia (CPEO) and Kearns-Sayre syndrome (KSS), with severe, early onset multi-organ failure^101^; the severity of dysphagia is linked to the extent of cricopharyngeal achalasia.^102^^,^^103^ In these disorders, impaired smooth muscle relaxation at the gastroesophageal sphincter may result in achalasia.^48^^,^^104^ The underlying cause of muscle weakness stems from impaired ATP production within mitochondria as a consequence of dysfunctional oxidative phosphorylation.^24^

### Myofibrillar Myopathies

Desminopathies, stemming from mutations in the desmin-encoding gene (*DES*), present with progressive bilateral distal muscle weakness that progresses to affect proximal muscles, accompanied by respiratory distress and cardiomyopathy.^19^ Mutations in the DES alpha-helical domain modulate the severity of manifestations, including dysphagia.^105^ Muscle weakness and dysfunction result from mutation-induced alterations in desmin structure and/or stability that maintains interfilamentous network integrity.^19^ A subset of desminopathies, alpha-B crystallinopathies, results from mutations in *CRYAB*-encoding alpha B-crystallin, a desmin-stabilizing chaperone protein.^19^ In addition to the distal muscle weakness characteristic of desminopathies, dysphonia and dysphagia are early symptoms.^55^^,^^56^ Like desmin, alpha B-crystallin is essential to maintain cytoskeletal structure in the sarcomere through its role as a chaperone for desmin and actin, and its multiple functions in homeostasis.^57^ Limb girdle dystrophy type 1A (LGMD1A), resulting from mutations in the myotilin gene (*MYOT*), is primarily characterized by progressive hip and shoulder muscle wasting, but may include nasal dysarthria resulting from oropharyngeal and facial muscle weakness.^59^^,^^60^ Mutations in myotilin, a sarcomeric assembly protein essential for sarcomere structural integrity, impairs effective muscle structure and function.^59^

### Muscular Dystrophies

Muscular dystrophies encompass a heterogeneous group of disorders characterized by progressive muscle degeneration. Duchenne muscular dystrophy (DMD) results from mutations in *DMD*, that leads to severe, progressive global muscle-wasting due to the loss of functional dystrophin expression.^106^ Multiple functions of this costameric protein are disrupted, including interactions with binding partners (eg, signaling proteins, cytoskeletal filaments), subsequently destabilizing myofiber structural integrity.^62^ *DMD* mutations resulting in partial dystrophin expression cause the milder Becker’s muscular dystrophy (BMD) phenotype.^107^ Although dysphagia in DMD can have a variable onset, it may worsen with disease progression, increasing the risk of malnutrition and aspiration.^63^^,^^64^ BMD classically has a milder disease progression, but some studies report individuals with BMD exhibiting comparable dysphagia severity to DMD, and even earlier dystrophic alterations in the oropharyngeal muscles involved in mastication.^66^^,^^67^

Autosomal dominant (and more rarely homozygous recessive) nucleotide-repeat expansion mutations in *PABPN1*, encoding polyadenylate-binding nuclear protein 1, are linked to oculopharyngeal muscular dystrophy (OPMD), a late-onset muscular dystrophy subtype. Characterized by progressive muscle weakness targeting muscles of the eyes, oropharynx cavity, and proximal limbs, individuals with OPMD present between the fourth to sixth decade of life with progressive ptosis, dysphagia, and neck and shoulder muscle weakness.^69^ Affected individuals may have deglutition and upper esophageal sphincter dysfunction with dysphagia-induced aspiration often requiring gastrostomy to maintain adequate nutrition.^69^^,^^108^ *In vitro* and *in vivo* studies reveal that OPMD-associated mutations promote nuclear aggregation and subsequent cellular dysfunction, but the exact mechanism remains elusive.^109^ There is limited understanding of how mutations in ubiquitously expressed PABPN1 lead to dystrophic changes in specific skeletal muscles, a question requiring further investigation.^109^

Myotonic dystrophy (DM) results from nucleotide expansion mutations, specifically in *DMPK* and *CMBP* resulting in DM1 and DM2 subtypes, respectively.^73^ In addition to myotonia, early-onset cataract, and muscle weakness, these subtypes commonly present with impaired swallowing, leading to choking, aspiration, and malnutrition.^73^, ^74^, ^75^ Imaging studies reveal the greatest impairment in the pharyngeal phase of swallowing,^110^ with evidence of decreased basal pressure and weakened contractions of the upper esophageal sphincter observed with esophageal manometry.^76^ Anorectal manometry and intrarectal sonography may uncover anal sphincter atrophy as the cause of fecal incontinence.^7^^,^^8^ The pathogenesis of DM1 and DM2 stems from aberrant RNA structure and sequestration of RNA-binding proteins highly expressed in skeletal muscle.^111^

Facioscapulohumeral dystrophy (FSHD) 1, a consequence of epigenetically induced toxic post-developmental DUX4 protein expression in myofibers, classically presents with winged scapulae and weak atrophic facial muscles.^112^ In affected individuals, these features contribute to mild to moderate impaired swallowing.^84^ Additionally, in FSHD, impaired cheek compression correlates with dysphagia and dysarthria.^113^ Although the loss and/or pathogenic epigenetic modifications of microsatellite repeats encoding *DUX4* leads to FSHD, the pathologic mechanisms of aberrant DUX4 expression remain under investigation.^85^

Non-myofibrillar LGMDs encompass a group of rare, heterogeneous clinical subtypes that manifest as progressive limb girdle muscle weakness; only a few are associated with GI symptoms. Progressive dysphagia and aspiration, resulting from compromised pharyngeal transit and esophageal dysmotility, are observed in LGMD2B, caused by mutations in the sarcolemma repair protein, dysferlin.^86^ Upper GI symptoms also occur in individuals affected by LGMD1D and LGMD2D, resulting from mutations in the ubiquitously expressed proteins encoded, respectively, by *DNAJB6* and *TNPO3*.^87^^,^^88^ Severe dysphagia may require placement of a percutaneous endoscopic gastrostomy tube to maintain feeding and nutrition.

### Miscellaneous

Early-onset myopathy, areflexia, respiratory distress, and dysphagia (EMARDD) results from mutations in *MEGF10*, a gene encoding a transmembrane protein expressed in neurons and skeletal muscle satellite cells, that impair proliferation and myogenesis.^89^ Affected individuals display severe early-onset muscle weakness, congenital myotonia, decreased fetal movements, finger contractures or equinovarus foot, and areflexia.^89^ These clinical pathologies further manifest in dysphagia and respiratory weakness, often leading to ventilation dependency and early death.^89^ Some *MEGF10* mutations result in milder variants (mvEMARDD) with attenuated muscle weakness and respiratory dysfunction; onset of symptoms can vary from congenital presentation to late-onset.^90^ Histologic abnormalities include multiple minicores, adipose infiltration, variable myofiber size, and fiber type I dominance.^90^, ^91^, ^92^ Some individuals have cleft, midline ridge, or high arched palates, and dysphagia leading to recurrent pulmonary infections due to aspiration.^90^, ^91^, ^92^

*ADSSL1* myopathy is a rare autosomal recessive disease with altered muscle-specific adenylosuccinate synthase, required for adenine nucleotide synthesis.^93^ This causes adipose infiltration of skeletal muscles, myofibrillar disorganization, internalized nuclei, variable myofiber size, rimmed vacuoles, increased connective tissue, inflammatory cell infiltrate, and sometimes nemaline bodies.^93^, ^94^, ^95^ In adolescence, affected individuals develop mild distal and/or proximal muscle atrophy and slowly progressive facial muscle weakness.^93^, ^94^, ^95^ Affected individuals commonly have a high-arched palate with dysphagia due to masticatory dysfunction and muscle fatigability.^93^^,^^95^

## Management of Heritable Myopathies

There are limited treatment options for individuals with inherited myopathies, particularly to manage the GI manifestations. Dysphagia is the most prevalent and debilitating GI complaint. To address feeding difficulties in infancy, nasogastric and gastrostomy tubes are commonly employed.^11^^,^^12^^,^^22^^,^^24^^,^^63^^,^^31^ Video fluoroscopy swallowing studies can evaluate impaired swallowing and ascertain aspiration risk.^96^^,^^75^ Other interventions, including injection of botulinum toxin into the cricopharyngeal muscle and cricopharyngeal dilatation, reportedly improved dysphagia in NM and OPMD, respectively.^70^^,^^114^ Individuals with XLMTM and DMD may require tracheostomy to manage severe dysphagia and recurrent aspiration.^63^ Dietary and nutritional assessments may identify the need for high-caloric diets or other interventions.^63^ Appetite stimulants may benefit individuals with MID and poor nutritional intake with minimally impaired swallowing.^48^ Speech therapy evaluation may help individuals with FSHD and those with impaired swallowing to develop compensatory strategies.^84^ Although a variety of approaches can help manage dysphagia associated with inherited myopathies, comprehensive individualized treatment plans require nutritional and dietary evaluation, radiographic studies of oropharyngeal bolus transfer, esophageal manometry, and others.

## Other Considerations Within the GI System

Notably, smooth muscle, extending from the mid-esophagus to the external anal sphincter, is also frequently affected in heritable myopathies as many of the described genes are expressed in both skeletal and smooth muscle. In desminopathies, DMs, and MIDs, vomiting, constipation, and small bowel diverticulosis may result from GI smooth muscle dysfunction.^48^^,^^49^, ^50^, ^115^ Individuals with DMD, DM, and MIDs are also reported to have intestinal pseudo-obstruction.^48^^,^^50^^,^^51^, ^77^, ^78^ Individuals with DMD, manifesting with abdominal pain and constipation, possibly because of pneumatosis cystoides intestinalis of the ascending colon, are reported.^27^ Although the underlying mechanisms remain obscure, patients with mitochondrial disorders are reported to be at risk for recurrent pancreatitis.^48^ Pancreatic inflammation may be subclinical with only biochemical or imaging abnormalities but is occasionally more severe, resulting in pancreatic insufficiency.^48^

## Acquired Myopathies

Although the focus of the current review is on the mechanisms underlying GI manifestations of heritable myopathies, acquired myopathies can also impact GI tract function. Inflammatory autoimmune myopathies, such as dermatomyositis, cause muscle inflammation and weakening. Dermatomyositis, characterized by cutaneous gottron papules and heliotrope rash, is associated with symmetrical proximal muscle weakness.^116^ GI muscular manifestations include dysphagia, dysphonia, and aspiration due to pharyngeal and proximal esophageal muscle weakness.^116^ Atrophy of muscle tissue, evidenced histologically by perivascular and perifascicular inflammation, includes perifascicular atrophy within muscle fibers.^117^ Like dermatomyositis, polymyositis presents with proximal symmetric myopathy, comparable histologic findings, and the potential for GI skeletal muscle involvement resulting in dysphagia.^118^ Levels of specific antibodies in polymyositis and dermatomyositis correlate with symptom severity (ie, dysphagia).^119^ Unfortunately, GI tract involvement portends a worse prognosis.^120^ Dermatomyositis and polymyositis are more common in women, and GI manifestations tend to occur more frequently at the extremes of age.^121^^,^^122^ Although corticosteroids remain the cornerstone of treatment for both diseases, particularly for active muscle disease in dermatomyositis, additional immunosuppressive therapies (eg, mycophenolate, methotrexate) may be necessary for long-term treatment.^118^^,^^123^^,^^124^ Distinct from dermatomyositis and polymyositis, inclusion body myositis has a more insidious onset and remains treatment-resistant,^121^ although pharyngoesophageal dilation may be beneficial.^125^, ^126^, ^127^

Infectious myopathies can also impact GI tract skeletal muscle, resulting in oropharyngeal dysfunction. Causes include bacterial (eg, *Staphylococcus*), parasitic (eg, *Trypanosoma cruzi*), fungal (eg, *Aspergillus*), and viral (eg, HIV) infections, with parasitic infection being the most likely to involve esophageal skeletal muscle.^128^, ^129^, ^130^ Larval encapsulations in the masseter and tongue muscles may result from *Trichinosis,* and megaesophagus may accompany inflammatory myositis from *T. cruzi* infiltration.^130^^,^^131^ Eosinophilic myositis of the tongue, pharynx, and larynx is reported in sarcocystosis.^132^ The risk of infectious myositis is increased in immunocompromised individuals.^130^

Rarely, toxic myopathy, involving GI skeletal muscle, is associated with statins and anti-malarial therapy.^133^ Reversible progressive dysphagia and muscle fatigue due to statin-induced HMG-CoA reductase necrotizing autoimmune myopathy is reported, but with substantial heterogeneity in the clinical manifestations.^134^^,^^135^ Although they can occur in isolation, toxic myopathies are more likely when drugs are prescribed in combination (eg, concurrent statin and fibrate intake).^136^ Drug discontinuation typically reverses myopathy, a better outcome than for other forms of acquired myopathy.

Metabolic abnormalities from hyper- and hypothyroidism can also induce skeletal myopathy. Rarely, the effects of hyperthyroidism on pharyngeal and proximal esophageal skeletal muscle cause dysphagia, regurgitation, and aspiration with pneumonia. Hypothyroidism can cause oropharyngeal dysphagia from decreased propagation velocity of skeletal muscle contraction, but smooth muscle can also contribute.^137^ Treating thyroid disorders resolves the related myopathies.^138^

## Discussion and Conclusions

Despite our comprehensive approach to reviewing the genetics and molecular biology of heritable myopathies, and their impact on gastrointestinal tract function, we acknowledge limitations. For example, we did not include dysphagia due to impaired neurological input to GI skeletal muscle as we considered this beyond the scope of the current review. Likewise, several primary skeletal myopathies were not included because they generally lack GI symptoms. Whereas several primary neuromuscular disorders, like neuromuscular junction disorders (eg, myasthenia gravis, Lambert-Eaton syndrome) and neurogenic diseases (eg, amyotrophic lateral sclerosis, spinal muscular atrophy) are associated with myopathic GI manifestations, because we focused on pure sarcomeric deficits, we also considered these beyond the scope of the present review.

As highlighted throughout this review, the primary GI manifestation of most skeletal myopathies is dysphagia. Nonetheless, little functional information is available to pinpoint specific oropharyngeal and/or esophageal muscle and segment involvement. We believe this presents unique opportunities for novel imaging and high-resolution manometric techniques to fill these knowledge gaps. A similar approach to studying anorectal function is likely to reveal subclinical manifestations. Moreover, increased access to high-throughput genome sequencing is likely to continue expanding the list of heritable myopathies. Lastly, potential reciprocal relationships between alterations in the gut microbiome and myopathic disorders may play a key role in clinical manifestations such as bloating, diarrhea, or malabsorption, and are worthy of further exploration.^139^, ^140^, ^141^, ^142^, ^143^ As individuals with heritable myopathies are geographically dispersed, future studies will require multi-center cooperation and coordination to define more precisely the interplay between skeletal myopathic mutations and gastrointestinal manifestations.
